# Clinical Outcomes and Prognosis Factors of Nivolumab Plus Chemotherapy or Multitarget Tyrosine Kinase Inhibitor in Multi-Line Therapy for Recurrent Hepatitis B Virus-Related Hepatocellular Carcinoma: A Retrospective Analysis

**DOI:** 10.3389/fonc.2020.01404

**Published:** 2020-08-26

**Authors:** Chao Chen, Li An, Ying Cheng, Xianwen Luo, Zixiong Li, Xiufeng Liu

**Affiliations:** ^1^Department of Medical Oncology of PLA Cancer Center, Jinling Hospital, Nanjing, China; ^2^Department of Gerontology, The Affiliated Zhongda Hospital of Southeast University, Nanjing, China

**Keywords:** hepatocellular carcinoma, hepatitis B virus, nivolumab, chemotherapy, tyrosine kinase inhibitor, programmed cell death protein 1

## Abstract

**Background:** This study investigates the potential predictors of nivolumab plus chemotherapy or multitarget tyrosine kinase inhibitor (TKI) treatment response in patients with recurrent hepatitis B virus (HBV)-related hepatocellular carcinoma (HCC).

**Methods:** Patients with recurrent hepatitis B virus-related HCC who underwent nivolumab plus chemotherapy or TKI treatment between July 2017 and June 2019 at Jinling Hospital in China were retrospectively evaluated and included in this study. These patients also had both complete medical charts and follow-up data available. Overall survival (OS) and progression-free survival (PFS) were calculated from the date of nivolumab initiation. Survival data were compared using log-rank tests, and the associations of patient characteristics with survival were estimated using Cox regression models.

**Results:** A total of 22 HCC patients were included in this cohort and constituted the basis for this analysis. Twenty progressed cases (91%) and 16 deaths (73%) were identified at a median follow-up of 8.8 months (range 1–25). The median OS from the time of nivolumab initiation was 10.7 months (95% CI, 0.8–20.6 months), with a median PFS of 5.1 months (95% CI, 3.1–7.0 months). The patients were divided into two risk groups according to a nomogram built by age, Eastern Cooperative Oncology Group (ECOG) status, hepatectomy status, and transarterial chemoembolization (TACE) use. The median PFS was 8.2 ± 2.8 months in the low-risk group compared with 1.9 ± 0.4 months in the high-risk group (*p* = 0.0018). The median OS was estimated as 16.8 ± 4.9 months for low-risk patients vs. 8.6 ± 3.5 months for high-risk patients (*p* = 0.13).

**Conclusion:** Nivolumab combined with chemotherapy or TKI treatment is effective in patients with recurrent hepatitis B virus-related HCC. It is observed that previous TACE treatment is associated with a better PFS, and worse PFS in those patients who received hepatectomy. Prospective studies are warranted to evaluate the effects of nivolumab combined chemotherapy or TKI on recurrent hepatitis B virus-related HCC.

## Introduction

Hepatocellular carcinoma (HCC) is currently ranked as the third leading cause of cancer-related mortality worldwide (1). In China, HCC has become the second leading cause of cancer-related deaths. The HCC mortality rate has been increasing, particularly in males aged 45 to 74 years old with chronic hepatitis B and hepatitis C viral infection, over recent decades (2). Hepatic resection remains the mainstay for curative treatment of HCC (3). However, long-term outcomes after resection remain unsatisfactory, with a high rate of recurrence of up to 60 to 70% within 5 years (1, 4, 5). Although guidelines have been published in the management of primary HCC, the management of recurrent HCC remains poorly defined.

The inhibition of both programmed cell death protein 1 (PD-1)/PD-1 ligand (PD-L1) and/or CTLA-4 signaling pathways by monoclonal antibodies (MAbs) to release the antitumor activity of preexisting tumor-specific T-cell immunity has initiated a new era for immunotherapy in oncology. Immune checkpoint inhibitors (ICPIs) (anti-PD-1 MAb such as nivolumab or pembrolizumab; anti-PD-L1 MAb such as atezolizumab, durvalumab, or avelumab; and CTLA-4 inhibitors such as ipilimumab or tremelimumab) have demonstrated a survival benefit and/or durable disease control in several advanced cancers. Nivolumab is an immune checkpoint inhibitor that blocks the interaction between programmed cell death protein 1 (PD-1) and its ligand PD-L1. Nivolumab has confirmed efficacy for the treatment of various tumor types (6–13). It is a fully human immunoglobulin (IgG4) monoclonal antibody inhibitor of PD-1 receptor, which has received accelerated US FDA approval in 2017 for advanced HCC patients who previously received sorafenib. Its safety and efficacy have been confirmed in the extensive cohort study of HCC patients, CheckMate 040 (NCT01658878). Nivolumab was associated with an improved median overall survival (OS) from 14.7 to 16.4 months in a randomized phase III study as the first-line setting of advanced HCC (NCT02576509) (14). Nivolumab has shown clinical antitumor activity in patients with advanced HCC. Nevertheless, studies have shown that compared to Sorafenib, OS and ORR improvement is seen in single Nivolumab treatment, but OS benefit cannot be concluded from these data. A prospective, randomized, controlled, international multicentered Phase III hepatocellular carcinoma study (EACH Study) initiated by our research team in 2013 showed that the median OS in the FOLFOX4 group, dominated by Oxaliplatin, had a median OS of 6.40 months, with an effective rate of 8.15%, and this study provides a new treatment option for patients with advanced hepatocellular carcinoma (15). To further improve the effectiveness of treatment in patients with liver cancer, nivolumab combined chemotherapy or multitargeted tyrosine kinase for hepatocellular carcinoma later line therapy was tried.

Many trials are underway to expand its application in different populations, as well as in combination approaches (16).

Consequently, we conducted a retrospective study to describe the clinical outcomes of nivolumab combined with chemotherapy or TKI treatment in patients with recurrent hepatitis B virus (HBV)-related HCC.

## Methods

### Patients

A total of 22 patients with recurrent hepatitis B virus-related HCC who started nivolumab treatment between July 2017 and June 2019 at Jinling Hospital in China were included in this study. We used the following inclusion criteria: All patients were recurrent hepatitis B virus-related HCC, aged 18 to 75 years, disease progression after sorafenib and lenvatinib in first-line treatment, Eastern Cooperative Oncology Group performance status (ECOG PS) 1–3, tumor base diameter >10 mm, and expected survival longer than 3 months, treatment period <3 months of nivolumab, or patients without follow-up.

Baseline and follow-up clinical data were collected retrospectively. The study was presented to, and approved by, the ethics committee of the hospital.

### Nivolumab Plus Chemotherapy or TKI Treatment

Patients received 3 mg/kg intravenous nivolumab every 3 weeks until disease progression, combined with TKIs (three patients treated with Sorafenib, eight patients treated with lenvatinib, and four patients treated with Regorafenib) in 15 patients and chemotherapy (oxaliplatin plus fluorouracil/leucovorin) in seven patients.

### Efficacy Assessment

Tumor response was evaluated using computed tomography or magnetic resonance imaging every two cycles (6 weeks) according to Response Evaluation Criteria in Solid Tumors (RECIST) guidelines, version 1.1. The efficacy was divided into complete response (CR), partial response (PR), stable disease (SD), and progressive disease (PD). The overall response rate (RR) was calculated by CR + PR, and the disease control rate (DCR) was calculated by CR + PR + SD. Adverse events were evaluated based on the National Cancer Institute Common Toxicity Criteria (NCI-CTC) version 4.0.

### Statistical Analysis

The patients were divided according to clinically meaningful cutoff values of factors using the ROC method. Survival curves were calculated by the Kaplan–Meier method, and univariate analysis was performed using the log-rank test. Factors with *p* < 0.1 on the univariate analysis were entered into the multivariate Cox regression model. Values of significance were set at *p* = 0.05. All analyses were performed using SPSS 21.0.

## Results

### Patient Characteristics

Patients' baseline and treatment information are shown in Table 1. The median age of the patients was 53 years (range: 36–71), and 19 of the 22 patients (86%) were male. The ECOG grade was 0–1 in 17 patients (77%) and 2–3 in 5 patients (23%). Thirteen patients (59%) were classified as Child–Pugh class A, and nine (41%) were classified as Child–Pugh class B. Eight (36%) patients underwent hepatectomy. Transarterial chemoembolization (TACE) was administered in 11 patients (50%).

**Table 1:** Demographic patient characteristics.

**Characteristic *N* = 22**	**No. (%)**
Median age (range), years	53 (36–71)
<63	17 (77%)
≥63	5 (23%)
Sex	
Male	19 (86%)
Female	3 (14%)
ECOG performance status (range)	1–3
<2	17 (77%)
≥2	5 (23%)
AFP, (range) ng/ml	1–68,368
<40	10 (45%)
≥40	12 (55%)
HBV-DNA, (range) copies/ml	0–7,300
<215	15 (68%)
≥215	7 (32%)
Child-Pugh	
A	13 (59%)
B	9 (41%)
Hepatectomy	
Yes	8 (36%)
No	14 (64%)
TACE	
Yes	11 (50%)
No	11 (50%)
Number of nivolumab cycles (range)	1–28
<9	13 (59%)
≥9	9 (41%)
Single dose of nivolumab, (range) mg	100–240
<200	3 (14%)
≥200	19 (86%)
Combined treatment	
Targeted therapy	15 (68%)
Chemotherapy	7 (32%)

### Clinical Outcome of Nivolumab Plus Chemotherapy or TKI Treatment

**Figure 1:**
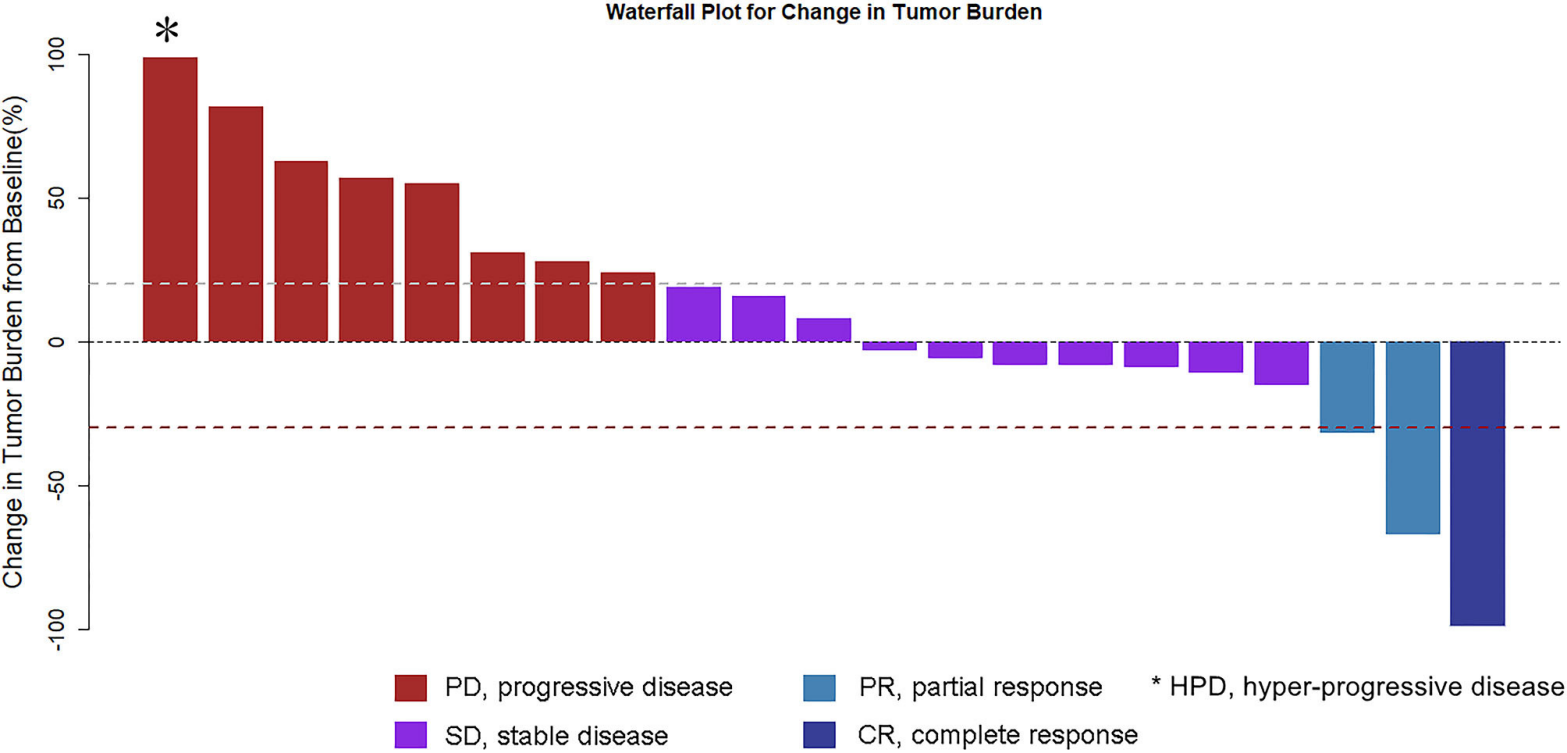
Waterfall plot for the best percentage change in target lesion size.

Complete imaging data of 21 patients were obtained. Patients were evaluated by spiral CT every 6 weeks: 1 (4.8%) case for CR, 2 (9.5%) cases for PR, 10 (47.6%) cases for SD, 8 (38%) cases for PD (including 1 case of hyper-progressive disease, HPD), RR 14.3%, DCR 61.9%. Tumor shrinkage is noted in Figure 1.

There were 20 cases of progression (91%) and 16 cases of deaths (73%) at a median follow-up of 8.8 months (range 1–25). The median OS from the time of nivolumab initiation was 10.7 months (95% CI, 0.8–20.6 months), with a median PFS of 5.1 months (95% CI, 3.1–7.0 months). In the univariate analysis, the following variables were found to be associated with prognosis: age, Child–Pugh grade, ECOG status, hepatectomy, TACE, and the number of nivolumab cycles (Table 2). The multivariate analysis retained the following independent prognostic factors for PFS: age [hazard ratio (HR) 0.12, *p* = 0.044], hepatectomy (HR 13.1, *p* = 0.009), TACE (HR 0.09, *p* = 0.004), and number of nivolumab cycles (HR 0.09, *p* = 0.010). In the multivariate analysis, only Child–Pugh grade was an independent predictor of OS (HR, 0.20, 95% CI 0.05 to 0.81, *p* = 0.024).

**Table 2:** Survival analysis of nivolumab treatment in patients with advanced/relapsed hepatitis B virus-related hepatocellular carcinoma.

	**Parameter**	**Univariate analysis**		**Multivariate analysis**	
		**HR (95% CI)**	***p***	**HR (95% CI)**	***p***
PFS	Gender (Male vs. Female)	0.50 (0.14–1.80)	0.286	0.35 (0.05–2.50)	0.294
	Age (≥63 vs. <63)	0.19 (0.04–0.84)	0.029	0.12 (0.02–0.94)	0.044
	HBV-DNA (≥215 vs. <215)	2.24 (0.84–5.93)	0.105	1.03 (0.34–3.07)	0.964
	AFP (≥40 vs. <40)	1.09 (0.42–2.84)	0.863	3.72 (0.48–28.54)	0.207
	Child-Pugh (A vs. B)	0.43 (0.16–1.13)	0.085	4.12 (0.76–22.40)	0.101
	ECOG (≥2 vs. <2)	2.87 (0.95–8.67)	0.061	6.88 (0.94–50.22)	0.057
	Hepatectomy (Yes vs. No)	2.85 (1.01–8.03)	0.048	13.10 (1.92–89.35)	0.009
	TACE (Yes vs. No)	0.42 (0.16–1.13)	0.085	0.09 (0.02–0.46)	0.004
	Number of nivolumab cycles (≥9 vs. <9)	0.28 (0.10–0.80)	0.017	0.09 (0.01–0.56)	0.010
	Single dose of nivolumab (≥200 vs. <200)	1.97 (0.45–8.65)	0.371	0.57 (0.06–5.42)	0.624
	Combined treatment (targeted therapy vs. chemo)	0.60 (0.21–1.74)	0.345	0.54 (0.11–2.61)	0.446
OS	Gender (Male vs. Female)	0.68 (0.14–3.21)	0.625	1.99 (0.27–10.31)	0.424
	Age (≥63 vs. <63)	0.17 (0.02–1.31)	0.089	0.16 (0.02–1.35)	0.092
	HBV-DNA (≥215 vs. <215)	2.21 (0.76–6.42)	0.143	1.62 (0.52–5.01)	0.406
	AFP (≥40 vs. <40)	1.60 (0.54–4.82)	0.398	2.33 (0.56–9.78)	0.248
	Child-Pugh (A vs. B)	0.21 (0.06–0.74)	0.015	0.20 (0.05–0.81)	0.024
	ECOG (≥2 vs. <2)	2.72 (0.77–9.69)	0.122	0.66 (0.13–3.32)	0.618
	Hepatectomy (Yes vs. No)	1.87 (0.61–5.81)	0.276	2.17 (0.59–7.95)	0.241
	TACE (Yes vs. No)	0.55 (0.19–1.61)	0.273	1.19 (0.30–4.75)	0.802
	Number of nivolumab cycles (≥9 vs. <9)	0.26 (0.07–0.93)	0.039	0.44 (0.12–1.61)	0.214
	Single dose of nivolumab (≥200 vs. <200)	4.21 (0.54–32.96)	0.171	0.80 (0.06–10.35)	0.863
	Combined treatment (targeted therapy vs. chemo)	1.02 (0.31–3.32)	0.970	1.03 (0.23–4.70)	0.968

**Figure 2:**
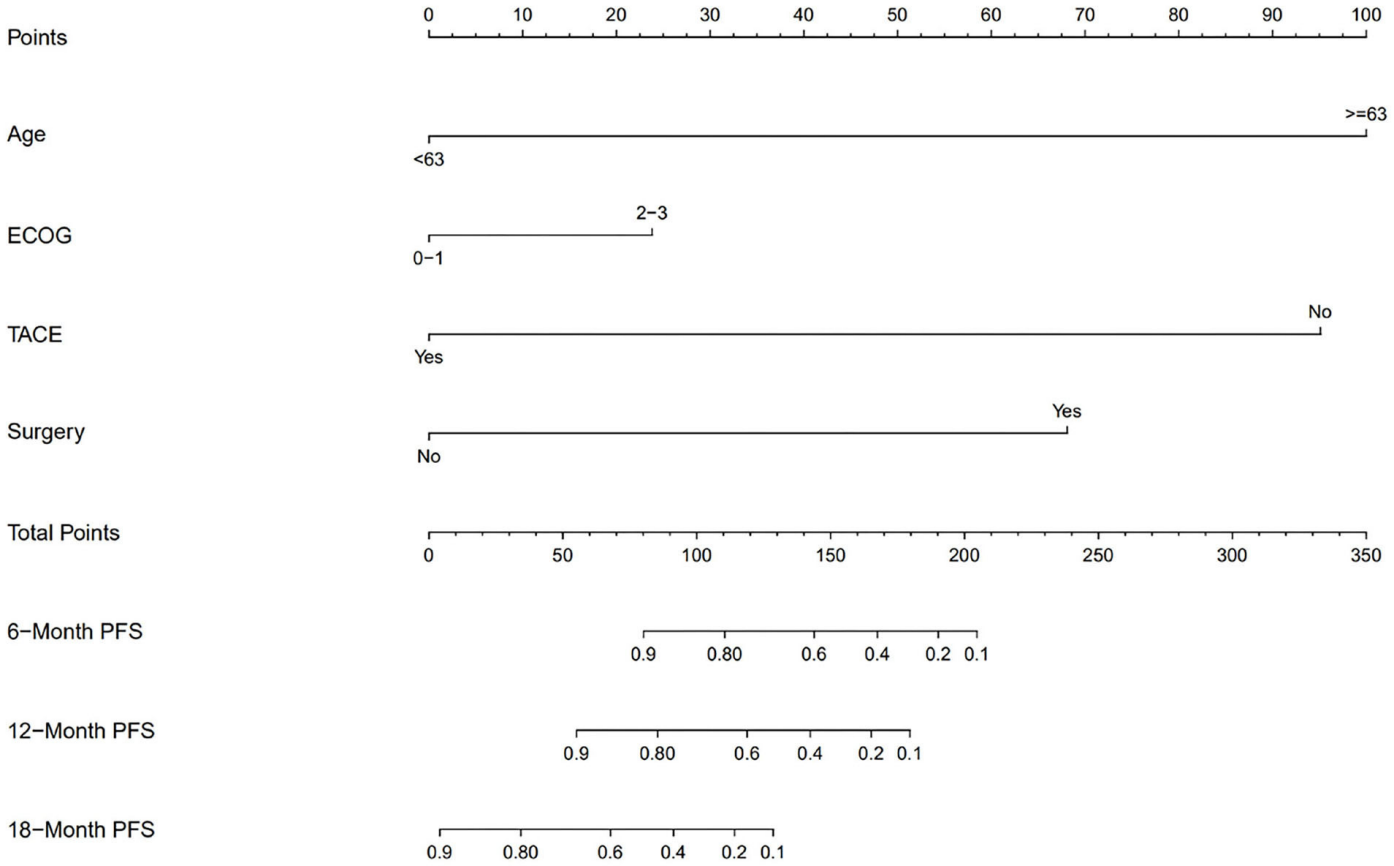
Nomogram for predicting the progression-free survival (PFS).

**Figure 3:**
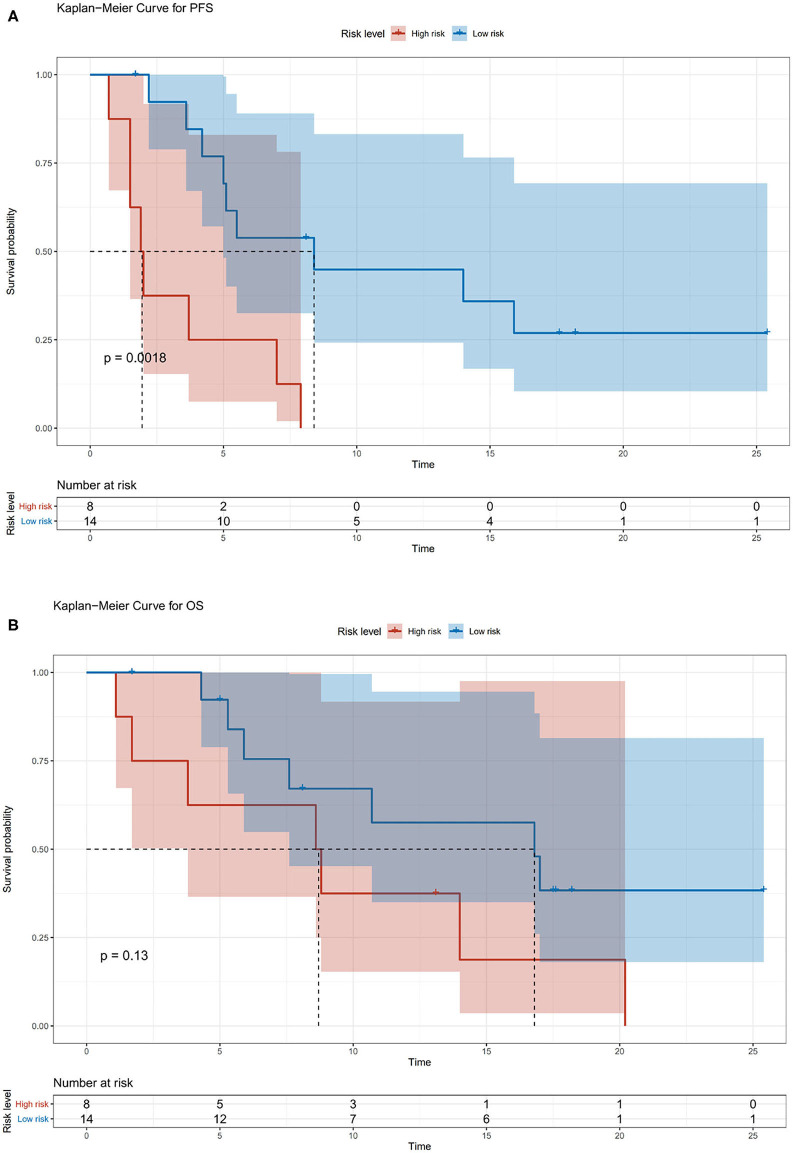
**(A)** Kaplan–Meier survival curves for risk stratification in the cohort PFS. **(B)** Kaplan–Meier survival curves for risk stratification in the cohort overall survival (OS).

We also divided the patients into two risk groups according to a nomogram (Figure 2) built by age, ECOG status, hepatectomy status, and TACE use. The median time of PFS was 8.2 ± 2.8 months in the low-risk group compared with 1.9 ± 0.4 months in the high-risk group (*p* = 0.0018; Figure 3A). The median time of OS was estimated at 16.8 ± 4.9 months for low-risk patients vs. 8.6 ± 3.5 months for high-risk patients (*p* = 0.13; Figure 3B).

### Adverse Events

Treatment-related AEs of any grade were less frequent. The majority of select AEs were grades 1 to 2, and the main adverse reactions included six cases of anorexia (27.3%), five cases of diarrhea (22.7%), four cases of hypothyroidism (18.2%), and two cases of hypophysitis (9.1%). The most frequently reported any-grade, treatment-related, select AE categories with nivolumab treatment were hypothyroidism (18.2%), diarrhea (9%), and hypophysitis (9%). Any grade and grade 3 or greater treatment-related serious events were reported in 4.5% and 1 of 22 patients, respectively. There is 4.5% of serious AE in upper gastrointestinal bleeding (Table 3). The immune-related adverse events (irAEs) are shown in Table 4.

**Table 3:** Common adverse events of clinical and laboratory abnormalities.

**Adverse event**	**Grade 1/2**		**Grade 3/4**		**Patients, *n* (%)**
	**Chemotherapy**	**TKIs treatment**	**Chemotherapy**	**TKIs treatment**	
**Non-hematologic**					
Hypertension	0	1	0	0	1 (4.5%)
Mucositis	0	1	0	0	1 (4.5%)
Hypothyroidism	1	2	0	1	4 (18.2%)
Fatigue	1	2	0	0	1 (4.5%)
Diarrhea	1	4	0	0	5 (22.7%)
Upper gastrointestinal bleeding	0	0	0	1	1 (4.5%)
Hypophysitis	1	1	0	0	2 (9.1%)
Anorexia	3	3	0	0	6 (27.3%)
Albuminuria	0	1	0	0	1 (4.5%)
Hyperbilirubinemia	0	1	0	0	1 (4.5%)
**Hematologic**					
Hemoglobin	0	0	0	1	1 (4.5%)
Leukocyte	0	0	1	0	1 (4.5%)
Platelets	0	0	1	0	1 (4.5%)

**Table 4:** Immune-related adverse events.

**Adverse event**	**Grade 1/2**		**Grade 3/4**		**Patients, *n* (%)**
	**Chemotherapy**	**TKIs treatment**	**Chemotherapy**	**TKIs treatment**	
Mucositis	0	1	0	0	1 (4.5%)
Hypothyroidism	1	2	0	1	4 (18.2%)
Fatigue	1	2	0	0	1 (4.5%)
Diarrhea	1	1	0	0	2 (9.1%)
Hypophysitis	1	1	0	0	2 (9.1%)
Anorexia	1	1	0	0	2 (9.1%)
Albuminuria	0	1	0	0	1 (4.5%)
Hyperbilirubinemia	0	1	0	0	1 (4.5%)

## Discussion

To our knowledge, this retrospective study is the first analysis of the efficacy of nivolumab treatment in patients with recurrent HBV-related HCC, which is a growing population with a poor prognosis in China. More than 70% of Chinese patients with HCC have HBV infection, whereas the majority of patients with HCC in Western developed countries have HCV infection (17). HCC patients with HBV infection are more prone to develop progressive diseases and have a poorer prognosis than HCC patients with HCV infection (18). The results showed that the median OS in this cohort was 10.7 months, which is higher than that in patients with recurrent HCC who received liver transplantation and were ineligible for surgical intervention; among these patients, median OS was reported to be 5 months (19). Eastern and Western HCC are highly heterogeneous; HCC patients in China are mainly hepatitis B virus-related, while in the CheckMate 459 study group of patients from China, only a total of 87 cases are mainly hepatitis B virus-related, with more non-hepatitis patients. Therefore, these patients are relatively less effective (15). This study showed that nivolumab displayed antitumor activity in recurrent HCC patients, even for a population with HBV infection.

Although immune checkpoint inhibitors can enhance the intrinsic tumor-suppressive microenvironment of the liver, another antitumor therapy is needed as a combination to enhance the induction of T-cell responses. Such combination treatment could result in a dramatic improvement in efficacy and clinical outcome in patients with HCC (20). Combinatorial therapies include checkpoint blockade immunotherapy with chemotherapy, targeted therapies, surgery, radiation therapy, or newer immunotherapies.

In our study, a nomogram was developed to predict the prognosis of patients with recurrent HCC based on four significant factors: age, ECOG status, hepatectomy status, and TACE use. Several studies have shown that the duration of survival is somewhat shorter in elderly patients than in younger patients (21, 22). In recurrent or metastatic squamous cell carcinoma of the head and neck, nivolumab resulted in a higher median OS in patients under 65 years old than in patients ≥65 years old (8.2 vs. 6.9 months) (23). ECOG has a significant influence on survival and facilitates physician selection of certain treatments (24). Nivolumab led to shorter OS in patients with previously treated advanced squamous non-small-cell lung cancer with ECOG PS 2 vs. 0–1 (25). Multiple overlapping signaling pathways are involved in liver regeneration and hepatocarcinogenesis, including Wnt/β-catenin and Notch (26). These signaling pathways play an important role in regulating the crosstalk between the different compartments of the tumor microenvironment (27, 28), which has been observed to correlate with the response to checkpoint blocking antibodies (29, 30). The Notch signaling pathway suppresses tumor-infiltrating CD8+ T-cell activity (31). A low level of tumor-infiltrating CD8+ T cells might be a promising prognostic factor of HCC, especially for Asian patients (32). Patients with HCC had a higher proportion of CD4 (+) CD25 (+) Tregs in peripheral blood (33). The proportion of Tregs in patients who were in stable condition or were improving after TACE decreased significantly, whereas the proportion of Tregs in patients who deteriorated increased significantly after TACE (33). Treg-induced inhibition of IFN-γ secretion can be partially blocked by PD-1 antibodies specifically in HCC patients (34). The nomogram was used to identify HCC patients who benefited from nivolumab combined chemotherapy or TKI treatment. Patients with low risk showed a significantly improved PFS (45% at 1 year, *P* = 0.0018) and a trend of improved OS (57% at 1 year, *P* = 0.13). In this study, no adverse dermatologic events were recorded, which is possibly due to the limited cases, and combined therapy might reduce the dermal toxicity.

The limitations of our study include, but are not limited to, the retrospective study and a small number of patients who were enrolled. According to multivariate analysis, due to the small sample size, several factors were associated with PFS with wide CIs, which reflected something not proper about the analysis methods. While RCTs remain the gold standard by which we base our treatment decisions, our retrospective analyses only provide important hypothesis-generating data, from which future practice-changing prospective trials can be built. The presence of tumor-infiltrating lymphocytes, expression of PD-L1, and tumor mutation burden within the liver were not tested to assess their roles in the response to immune checkpoint inhibition with nivolumab in our study. Larger and prospective clinical studies are needed to determine the most effective duration of immunotherapy combined TKI therapy and the best predictive biomarkers of response and to correlate the response. Combination therapy with checkpoint blockade is being investigated across a diverse range of tumor types and settings, including phase three trials (ClinicalTrials.gov numbers NCT01658878, NCT03439891, NCT03211416, NCT03418922, NCT03006926, NCT03347292, NCT01658878, NCT03299946, and NCT03289533).

In conclusion, nivolumab combined chemotherapy or TKI treatment is effective for patients with recurrent hepatitis B virus-related hepatocellular carcinoma; however, further research efforts are essential to confirm our data.

## Data Availability Statement

The original contributions presented in the study are included in the article/supplementary material, further inquiries can be directed to the corresponding author/s.

## Ethics Statement

The studies involving human participants were reviewed and approved by the Ethics Committee of Jinling Hospital. The patients/participants provided their written informed consent to participate in this study.

## Author Contributions

All authors listed have made a substantial, direct and intellectual contribution to the work, and approved it for publication.

## Conflict of Interest

The authors declare that the research was conducted in the absence of any commercial or financial relationships that could be construed as a potential conflict of interest.
